# SUPPORT AND EDUCATION NEED FULFILLMENT IN INFORMAL DEMENTIA CAREGIVERS

**DOI:** 10.1093/geroni/igz038.1056

**Published:** 2019-11-08

**Authors:** Nicholas James, Daniel Paulson

**Affiliations:** University of Central Florida, Orlando, Florida, United States

## Abstract

Caregivers vary widely in their need for and utilization of support services, and there are many reasons for this (e.g., time or financial cost, distance, perception of need). This study explored the disparity between services that are desired and those that are utilized, and examined the hypothesis that unmet needs are a determinant of caregiver burden. An online sample of informal caregivers (N=151) responded to a questionnaire containing a list of common caregiver support services: sharing duties, professional transportation, respite care, non-profit community organizations, financial counseling, caregiver education programs, support groups. Participants were presented with a list of support services and information and asked to indicate A) which they desired and B) which they had received while providing care. Respite care and financial counseling were identified as support that caregivers were unable to obtain, while caregiving education and family/friend support were most commonly fulfilled. A linear regression model controlling for demographic variables was constructed. Unfulfilled support needs accounted for 40.6% of variance in caregiver burden, however the final model included only total ratio of unfulfilled services, transportation services, stress management skills, professional treatment for the caregiver, and behavioral management skills. Results highlight the unique contribution of certain support services in burden reduction. These findings imply a need to improve accessibility to caregiver support, especially those which require payment (e.g., transportation aids and mental healthcare). Further implications and actionable changes related to caregiver support services are discussed.

